# Supplementary material for: Adjusting for allometric scaling in ABIDE I challenges subcortical volume differences in autism spectrum disorder
Source: Hum Brain Mapp. 2020 Jul 30;41(16):4610–29. doi: 10.1002/hbm.25145 (PMC7555078; doi:10.1002/hbm.25145)
Supplement: Supplementary file 1 — Data S1 Supporting Information. [file HBM-41-4610-s001.docx]

Supplementary Materials

Table of Contents

[1. Demographics by Scanner Sites. 2](#_Toc26790790)

[2. Power Analyses 17](#_Toc26790791)

[3. MGCFA & LMEMs Assumptions 18](#_Toc26790792)

[4. Replication of Zhang and Colleagues (2018): Methodology 23](#_Toc26790793)

[5. MGCFA Results 25](#_Toc26790794)

[6. Replication Results 30](#_Toc26790795)

1. **Demographics by Scanner Sites.**

The distribution of participant brain volumes, age, sex, FSIQ, and number of participants somewhat differed between scanner sites and varied between the present study and Zhang and coleagues (2018; e.g. Carnegie Melon scanner site participants were not included in the present study). Considering that a number of participants were removed from the analyses following segmentation quality checks, we examined if the remaining ASD individuals were still matched by age, sex, FSIQ, and scanner sites to controls. A total of 302 ASD participants were matched by sex, age, sites, and FSIQ to 302 control participants (MatchIt R package; Ho, Imai, King, & Stuart, 2011) and the remaining 50 unmatched controls were included in the analyses to increase the sample size.

Participants did not differ in across sites in terms of handedness (χ^2^ _(12)_= 14.8, p = 0.206) although they differed in sex ratio (χ2 _(14)_= 43.9, p < 0.001), Full Scale Intelligence Quotient (FSIQ) scores (χ^2^ _(14)_= 34.5, p = 0.002), and age (χ^2^_(14)_= 250.5, p < 0.001, d = 1.53). The distribution of all brain volumes, age, and FSIQ of Autism Spectrum Disorder (ASD) and control participants differed by scanner site following visual inspection and varied from the distribution of participants by scanner sites as shown by Figures S1-13. The figures correspond to the same figures included in Zhang and colleagues 's (2018) supplemental information to facilite sample comparison between our study and Zhang and colleagues (2018). Zhang and colleagues’ (2018) supplementary figures are available [here](https://www.cambridge.org/core/journals/psychological-medicine/article/revisiting-subcortical-brain-volume-correlates-of-autism-in-the-abide-dataset-effects-of-age-and-sex/CB66FFA7347DBE59C446BA66B1BA1A66#fndtn-supplementary-materials).


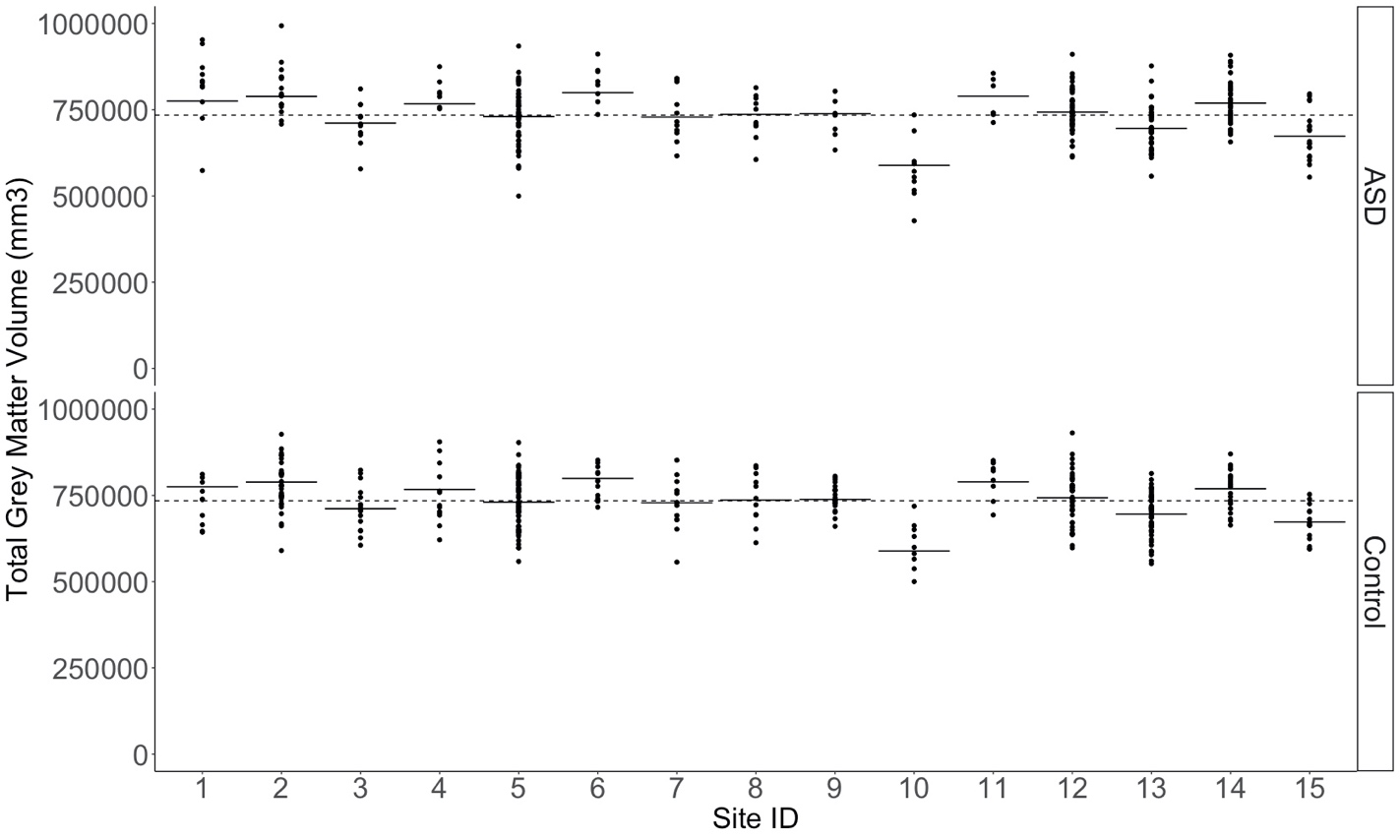


**Figures S1. Distribution of Total Grey Matter Volumes of Autism Spectrum Disorder (ASD) and Control Participants by Scanner Site**. Circles represent the brain volume of each participant. (1 = California Institute of Technology; 2 = Kenny Krieger Institute; 3 = University of Leuven Sample; 4= Ludwig Maximilians University Munich; 5 = NYU Langone Medical Center; 6 = Oregon Health and Science University; 7= Olin, Institute of Living at Hartford Hospital; 8= University of Pittsburgh School of Medicine; 9 = San Diego State University; l0 = Stanford University; 11 = Trinity Center for Health Sciences; 12 = University of California Los Angeles; 13 = University of Michigan Sample; 14 = University of Utah School of Medicine; 15 = Yale Child Study Center).


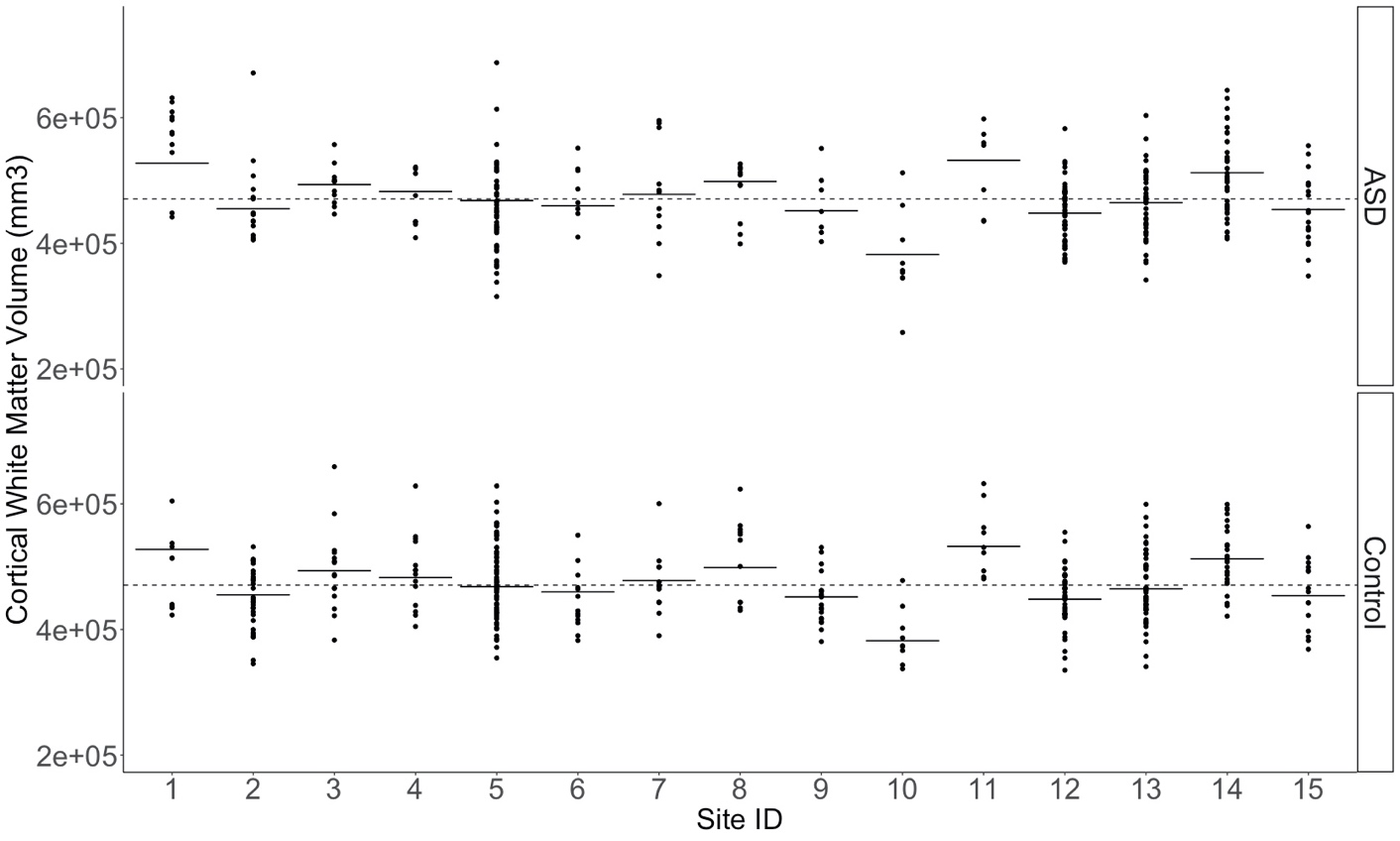


**Figures S2. Distribution of Cortical White Matter Volumes of Autism Spectrum Disorder (ASD) and Control Participants by Scanner Site**. Circles represent the brain volume of each participant. (1 = California Institute of Technology; 2 = Kenny Krieger Institute; 3 = University of Leuven Sample; 4= Ludwig Maximilians University Munich; 5 = NYU Langone Medical Center; 6 = Oregon Health and Science University; 7= Olin, Institute of Living at Hartford Hospital; 8= University of Pittsburgh School of Medicine; 9 = San Diego State University; l0 = Stanford University; 11 = Trinity Center for Health Sciences; 12 = University of California Los Angeles; 13 = University of Michigan Sample; 14 = University of Utah School of Medicine; 15 = Yale Child Study Center).


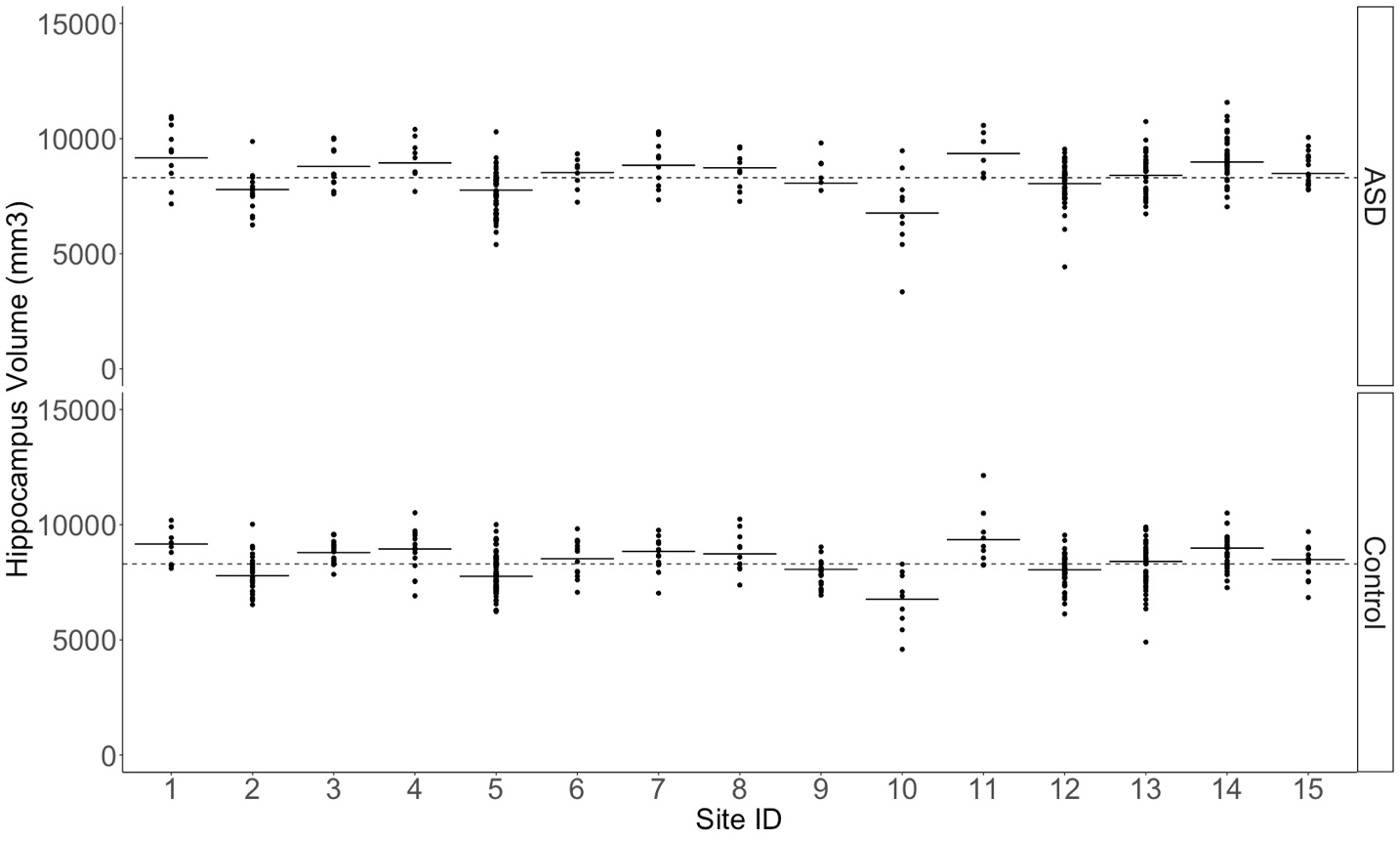


**Figures S3. Distribution of Hippocampal Volumes of Autism Spectrum Disorder (ASD) and Control Participants by Scanner Site**. Circles represent the brain volume of each participant. (1 = California Institute of Technology; 2 = Kenny Krieger Institute; 3 = University of Leuven Sample; 4= Ludwig Maximilians University Munich; 5 = NYU Langone Medical Center; 6 = Oregon Health and Science University; 7= Olin, Institute of Living at Hartford Hospital; 8= University of Pittsburgh School of Medicine; 9 = San Diego State University; l0 = Stanford University; 11 = Trinity Center for Health Sciences; 12 = University of California Los Angeles; 13 = University of Michigan Sample; 14 = University of Utah School of Medicine; 15 = Yale Child Study Center).


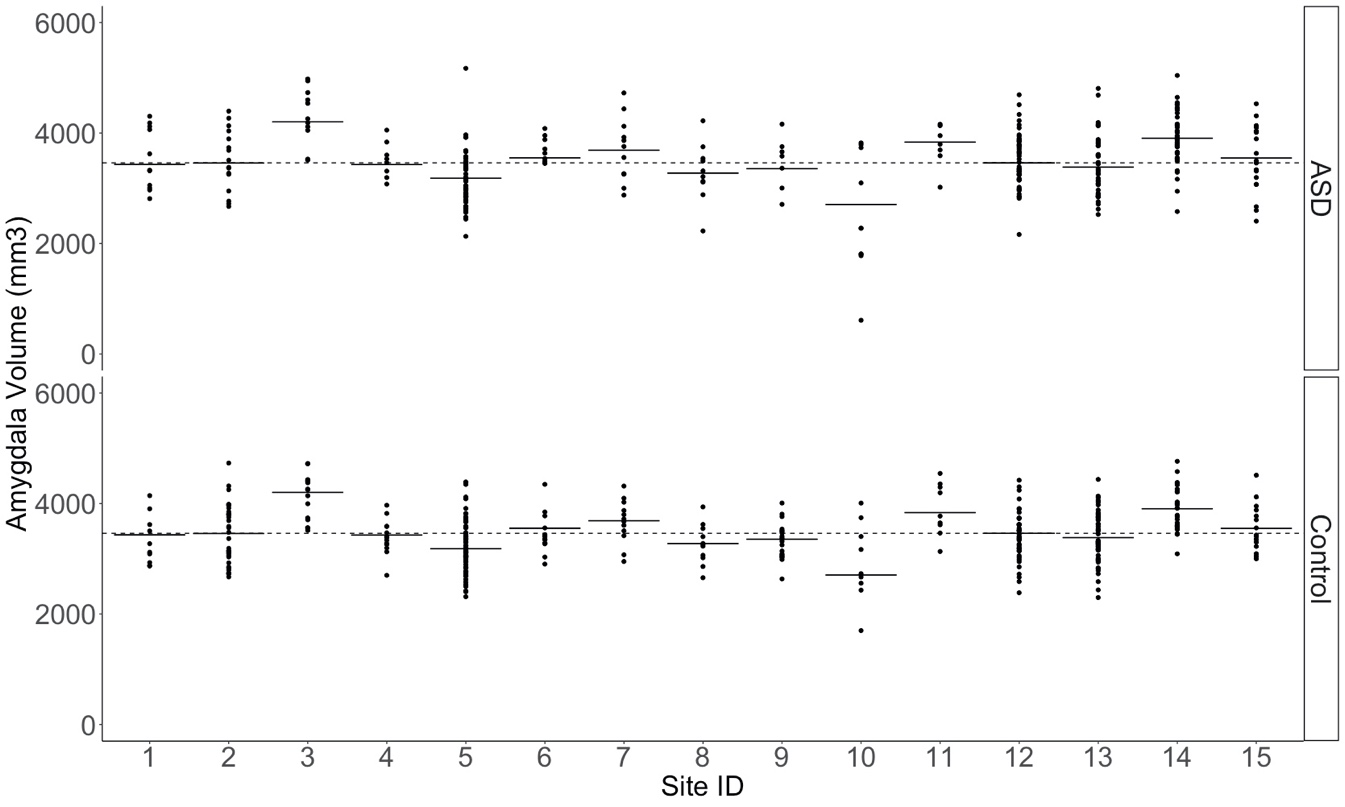


**Figures S4. Distribution of the Amygdala Volumes of Autism Spectrum Disorder (ASD) and Control Participants by Scanner Site**. Circles represent the brain volume of each participant. (1 = California Institute of Technology; 2 = Kenny Krieger Institute; 3 = University of Leuven Sample; 4= Ludwig Maximilians University Munich; 5 = NYU Langone Medical Center; 6 = Oregon Health and Science University; 7= Olin, Institute of Living at Hartford Hospital; 8= University of Pittsburgh School of Medicine; 9 = San Diego State University; l0 = Stanford University; 11 = Trinity Center for Health Sciences; 12 = University of California Los Angeles; 13 = University of Michigan Sample; 14 = University of Utah School of Medicine; 15 = Yale Child Study Center).


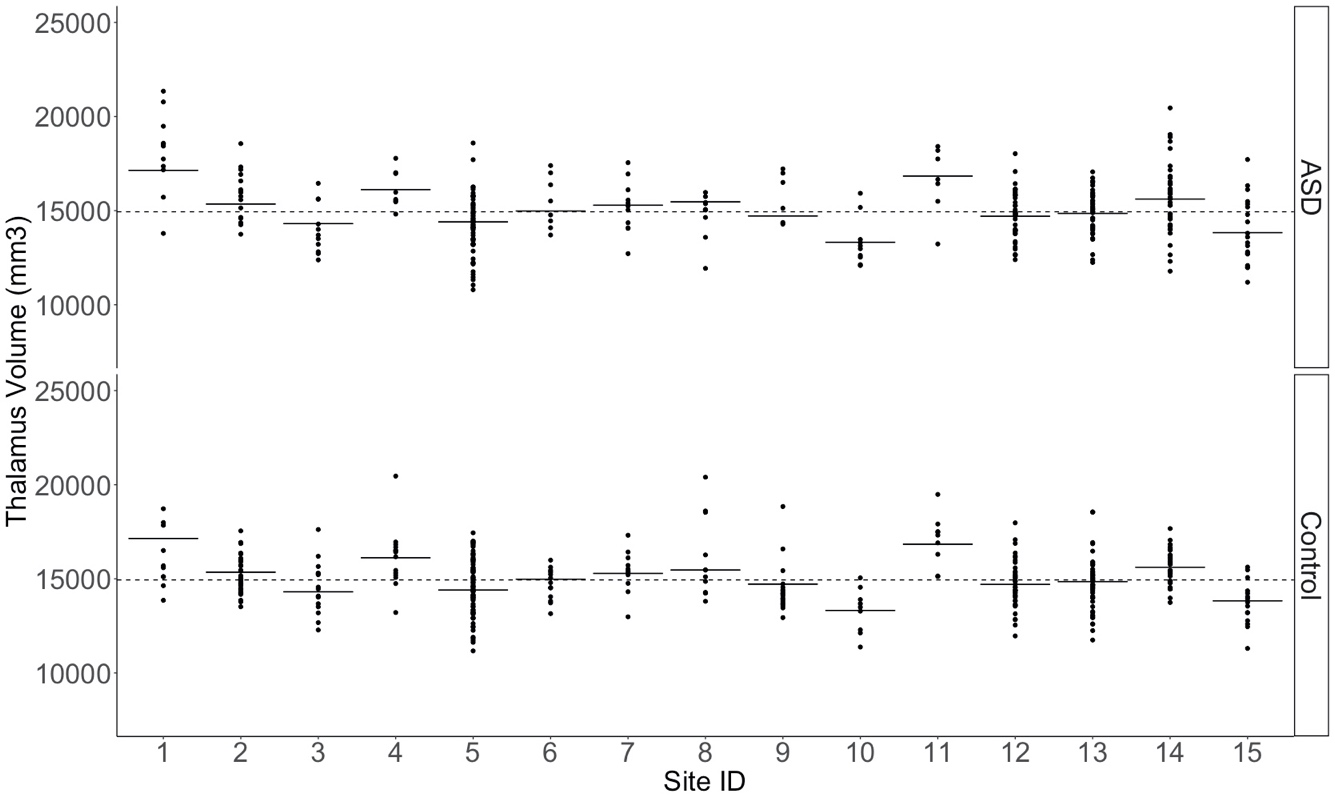


**Figures S5. Distribution of Thalamic Volumes of Autism Spectrum Disorder (ASD) and Control Participants by Scanner Site**. Circles represent the brain volume of each participant. (1 = California Institute of Technology; 2 = Kenny Krieger Institute; 3 = University of Leuven Sample; 4= Ludwig Maximilians University Munich; 5 = NYU Langone Medical Center; 6 = Oregon Health and Science University; 7= Olin, Institute of Living at Hartford Hospital; 8= University of Pittsburgh School of Medicine; 9 = San Diego State University; l0 = Stanford University; 11 = Trinity Center for Health Sciences; 12 = University of California Los Angeles; 13 = University of Michigan Sample; 14 = University of Utah School of Medicine; 15 = Yale Child Study Center).


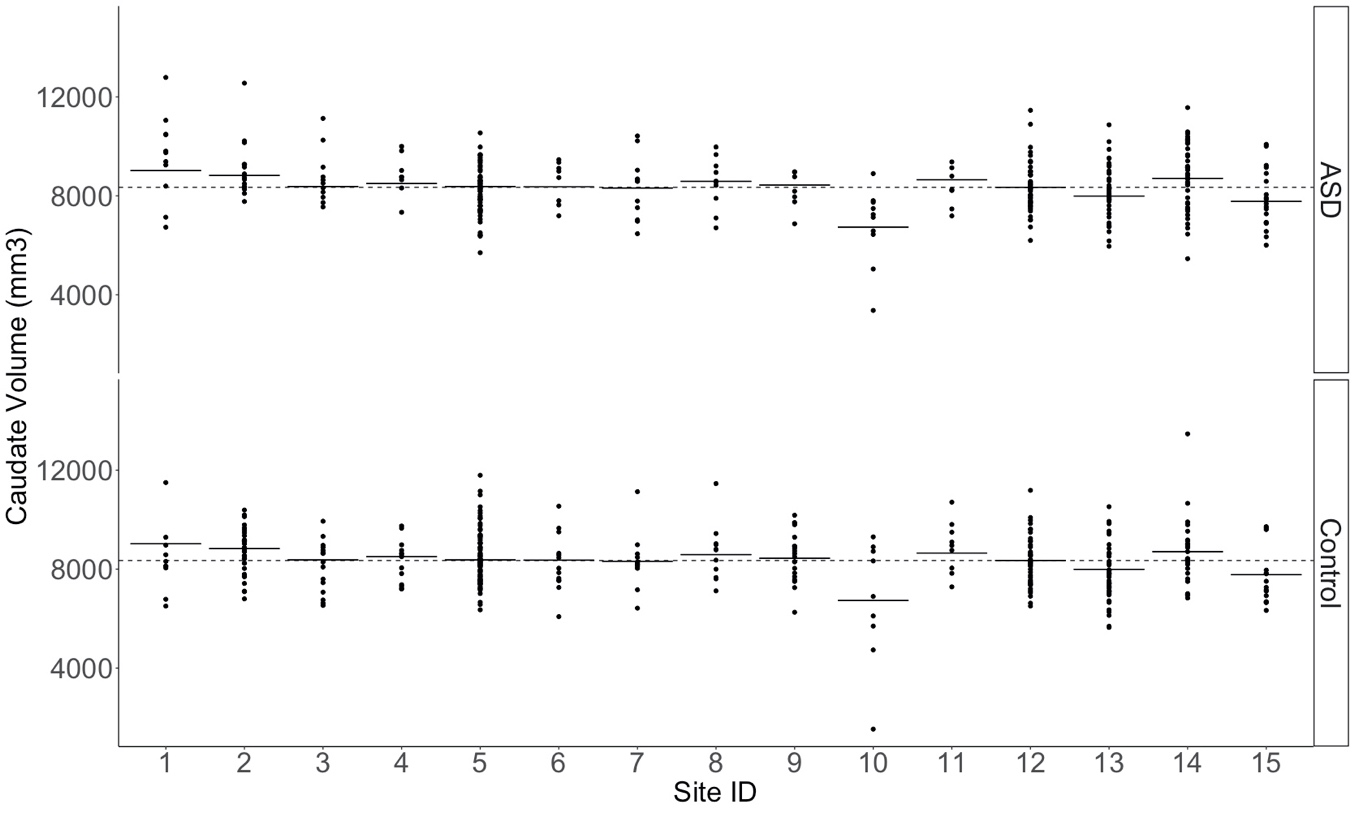


**Figures S6. Distribution of Caudate Volumes of Autism Spectrum Disorder (ASD) and Control Participants by Scanner Site**. Circles represent the brain volume of each participant. (1 = California Institute of Technology; 2 = Kenny Krieger Institute; 3 = University of Leuven Sample; 4= Ludwig Maximilians University Munich; 5 = NYU Langone Medical Center; 6 = Oregon Health and Science University; 7= Olin, Institute of Living at Hartford Hospital; 8= University of Pittsburgh School of Medicine; 9 = San Diego State University; l0 = Stanford University; 11 = Trinity Center for Health Sciences; 12 = University of California Los Angeles; 13 = University of Michigan Sample; 14 = University of Utah School of Medicine; 15 = Yale Child Study Center).


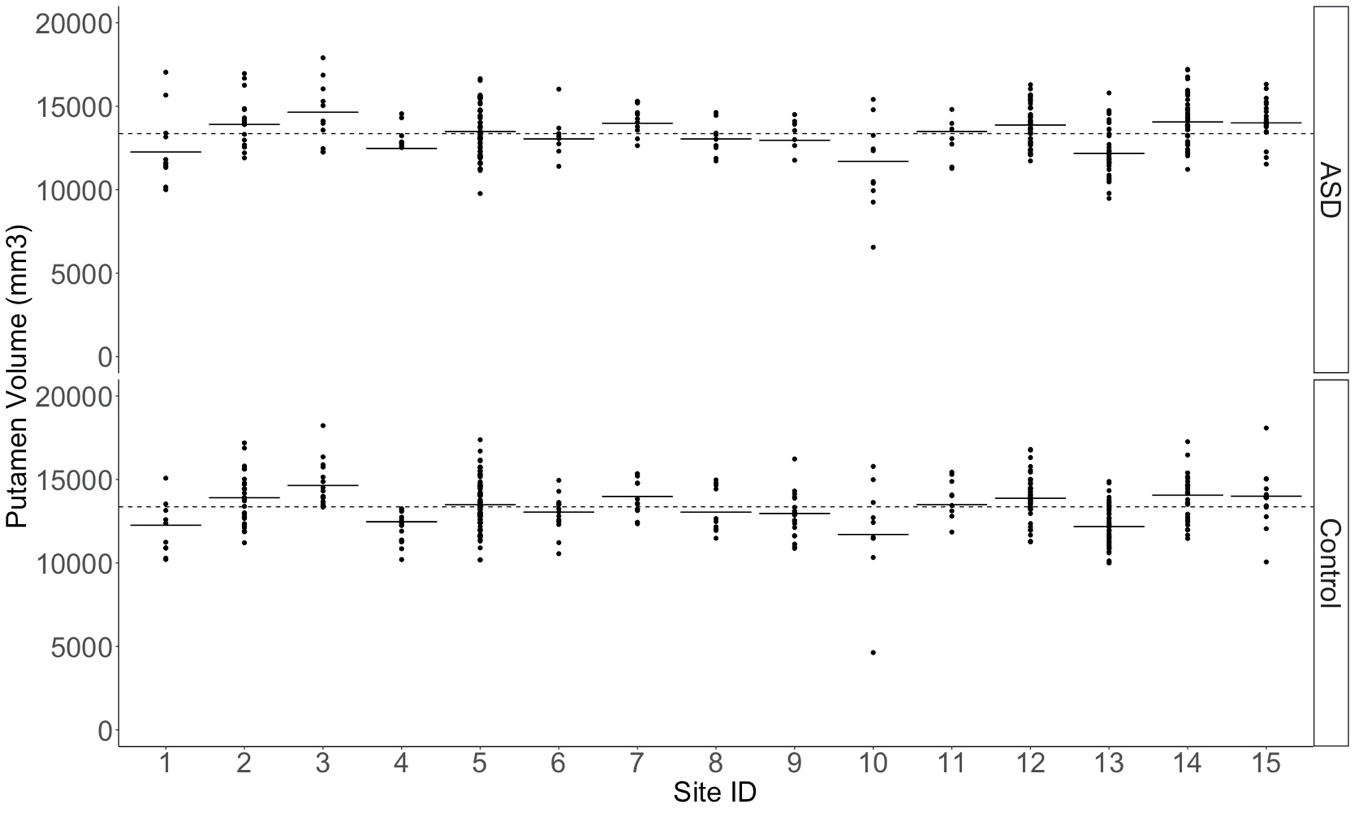


**Figures S7. Distribution of Putamen Volumes of Autism Spectrum Disorder (ASD) and Control Participants by Scanner Site**. Circles represent the brain volume of each participant. (1 = California Institute of Technology; 2 = Kenny Krieger Institute; 3 = University of Leuven Sample; 4= Ludwig Maximilians University Munich; 5 = NYU Langone Medical Center; 6 = Oregon Health and Science University; 7= Olin, Institute of Living at Hartford Hospital; 8= University of Pittsburgh School of Medicine; 9 = San Diego State University; l0 = Stanford University; 11 = Trinity Center for Health Sciences; 12 = University of California Los Angeles; 13 = University of Michigan Sample; 14 = University of Utah School of Medicine; 15 = Yale Child Study Center).


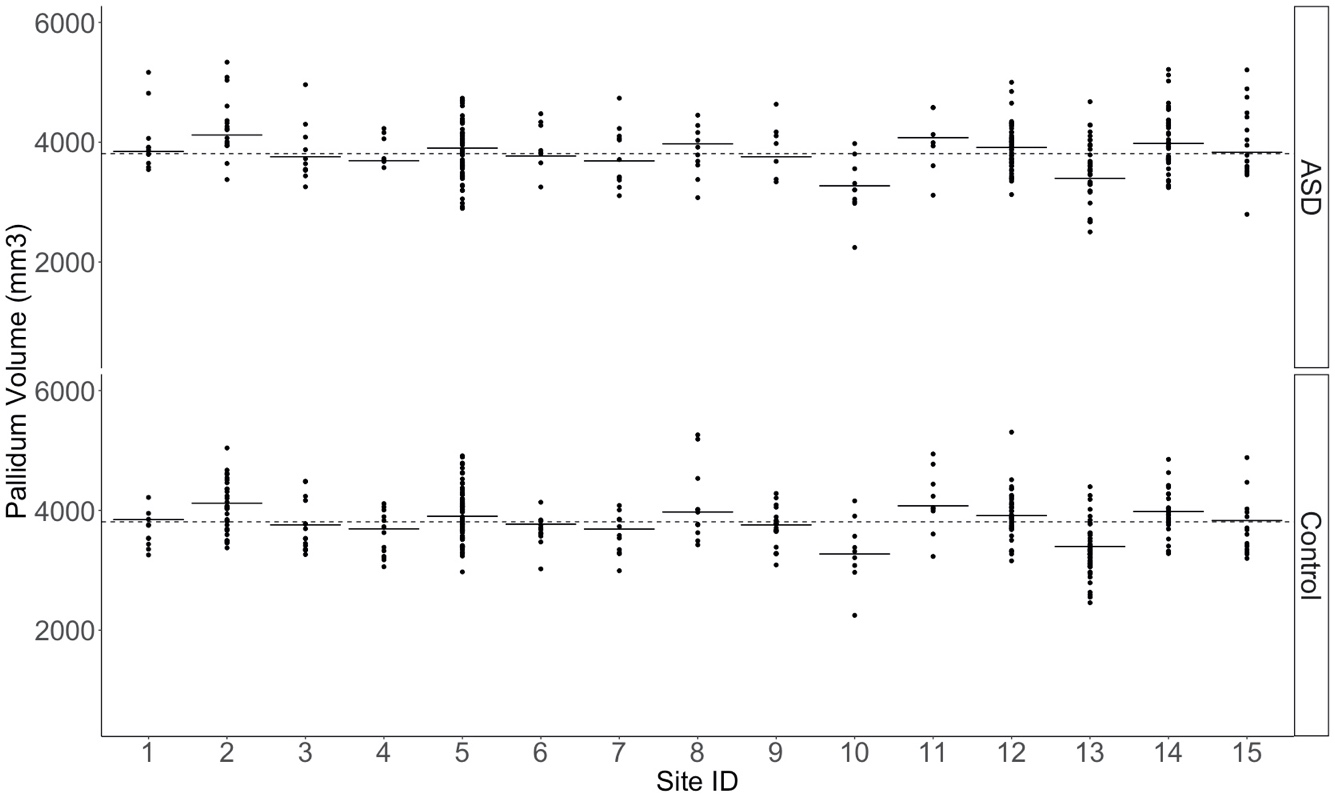


**Figures S8. Distribution of Pallidum Volumes of Autism Spectrum Disorder (ASD) and Control Participants by Scanner Site**. Circles represent the brain volume of each participant. (1 = California Institute of Technology; 2 = Kenny Krieger Institute; 3 = University of Leuven Sample; 4= Ludwig Maximilians University Munich; 5 = NYU Langone Medical Center; 6 = Oregon Health and Science University; 7= Olin, Institute of Living at Hartford Hospital; 8= University of Pittsburgh School of Medicine; 9 = San Diego State University; l0 = Stanford University; 11 = Trinity Center for Health Sciences; 12 = University of California Los Angeles; 13 = University of Michigan Sample; 14 = University of Utah School of Medicine; 15 = Yale Child Study Center).


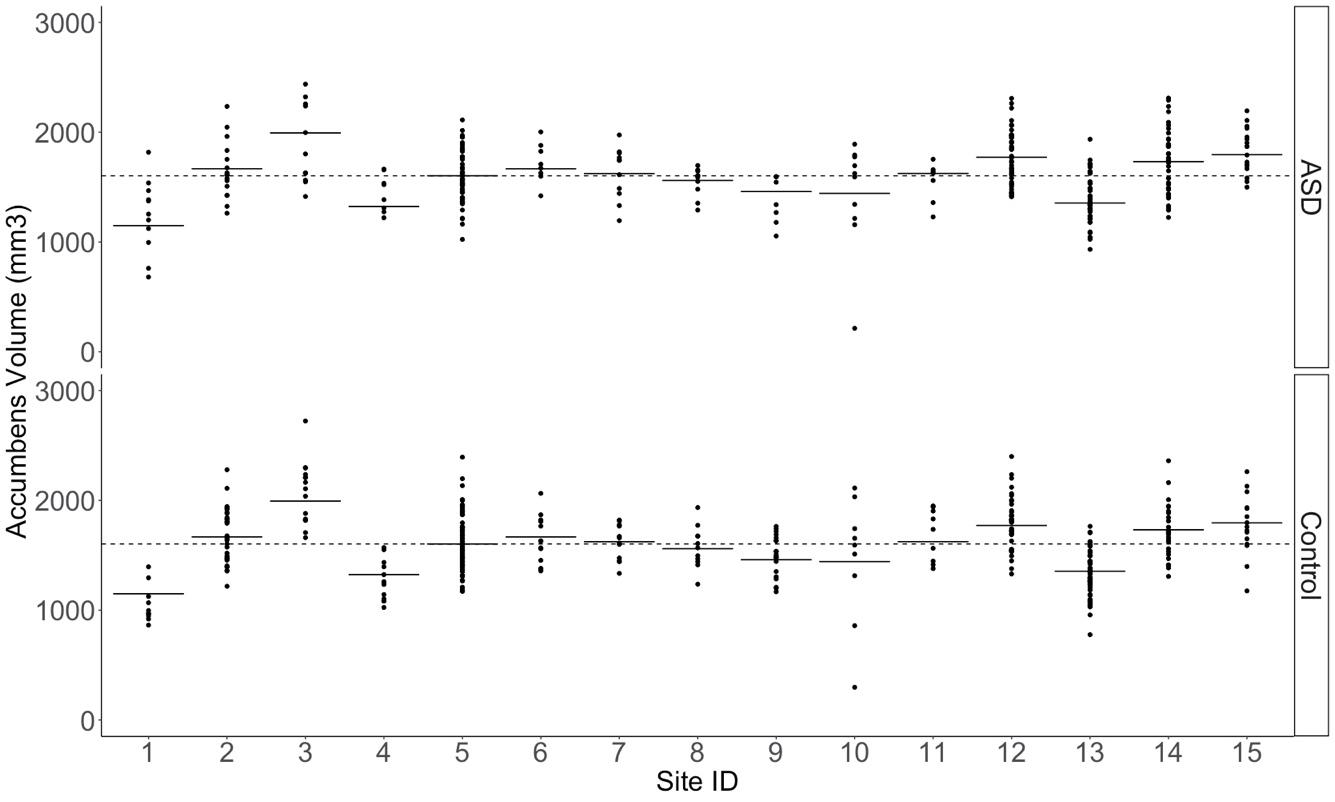


**Figures S9. Distribution of the Accumbens Volumes of Autism Spectrum Disorder (ASD) and Control Participants by Scanner Site**. Circles represent the brain volume of each participant. (1 = California Institute of Technology; 2 = Kenny Krieger Institute; 3 = University of Leuven Sample; 4= Ludwig Maximilians University Munich; 5 = NYU Langone Medical Center; 6 = Oregon Health and Science University; 7= Olin, Institute of Living at Hartford Hospital; 8= University of Pittsburgh School of Medicine; 9 = San Diego State University; l0 = Stanford University; 11 = Trinity Center for Health Sciences; 12 = University of California Los Angeles; 13 = University of Michigan Sample; 14 = University of Utah School of Medicine; 15 = Yale Child Study Center).


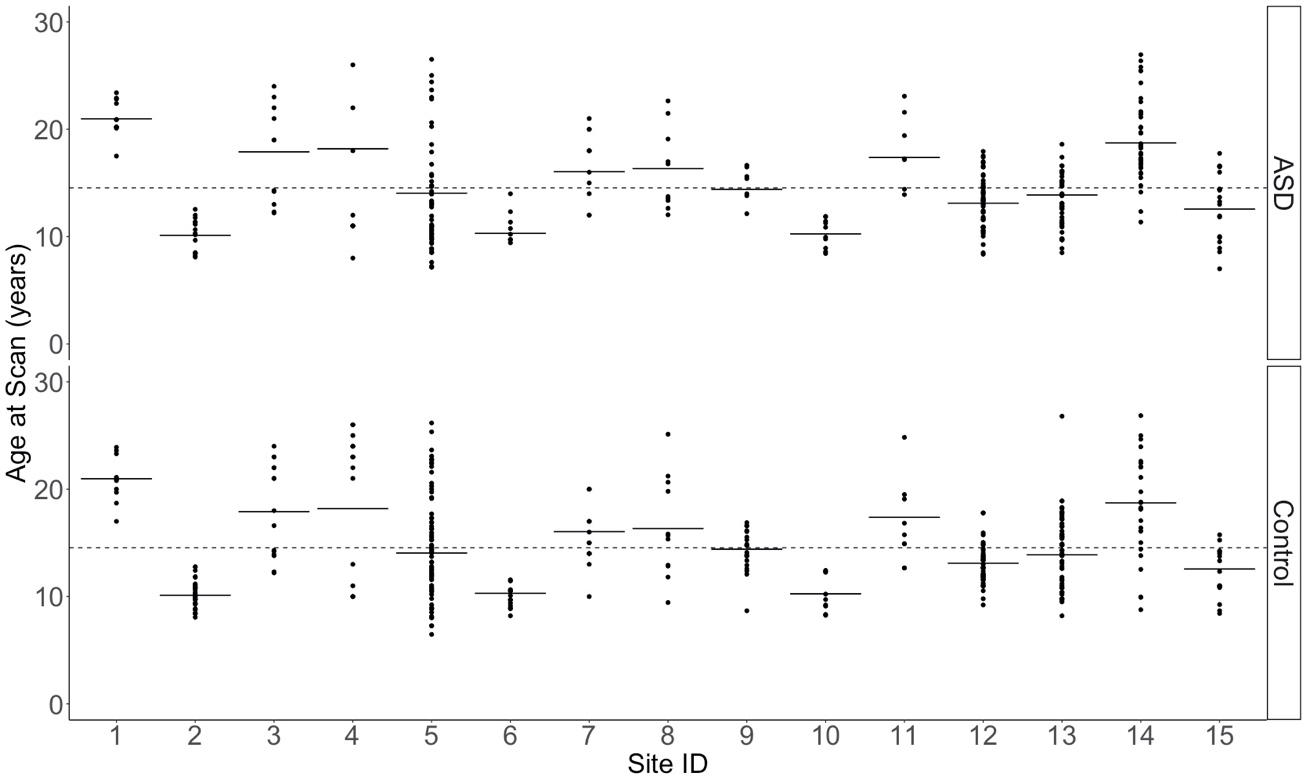


**Figures S10. Distribution of Age of Autism Spectrum Disorder (ASD) and Control Participants by Scanner Site**. Circles represent the brain volume of each participant. (1 = California Institute of Technology; 2 = Kenny Krieger Institute; 3 = University of Leuven Sample; 4= Ludwig Maximilians University Munich; 5 = NYU Langone Medical Center; 6 = Oregon Health and Science University; 7= Olin, Institute of Living at Hartford Hospital; 8= University of Pittsburgh School of Medicine; 9 = San Diego State University; l0 = Stanford University; 11 = Trinity Center for Health Sciences; 12 = University of California Los Angeles; 13 = University of Michigan Sample; 14 = University of Utah School of Medicine; 15 = Yale Child Study Center).


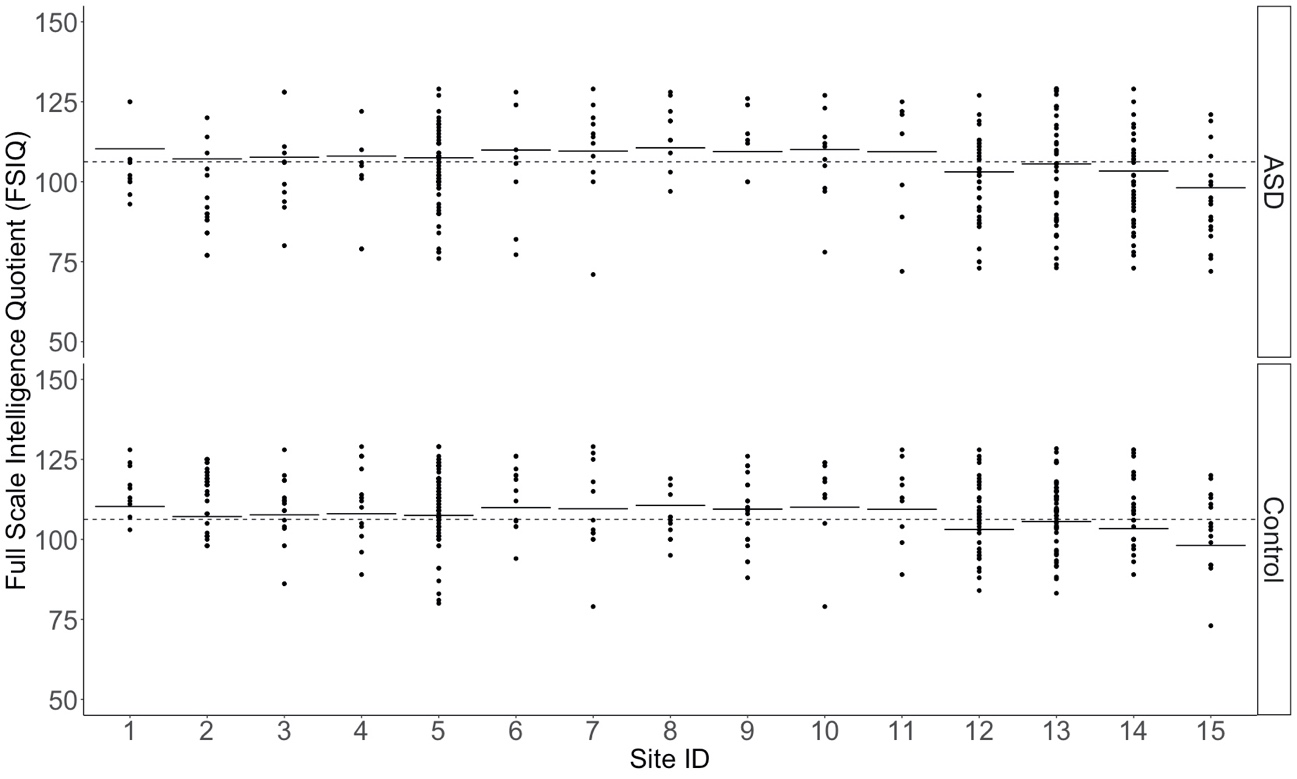


**Figures S11. Distribution of FSIQ of Autism Spectrum Disorder (ASD) and Control Participants by Scanner Site**. Circles represent the brain volume of each participant. (1 = California Institute of Technology; 2 = Kenny Krieger Institute; 3 = University of Leuven Sample; 4= Ludwig Maximilians University Munich; 5 = NYU Langone Medical Center; 6 = Oregon Health and Science University; 7= Olin, Institute of Living at Hartford Hospital; 8= University of Pittsburgh School of Medicine; 9 = San Diego State University; l0 = Stanford University; 11 = Trinity Center for Health Sciences; 12 = University of California Los Angeles; 13 = University of Michigan Sample; 14 = University of Utah School of Medicine; 15 = Yale Child Study Center).


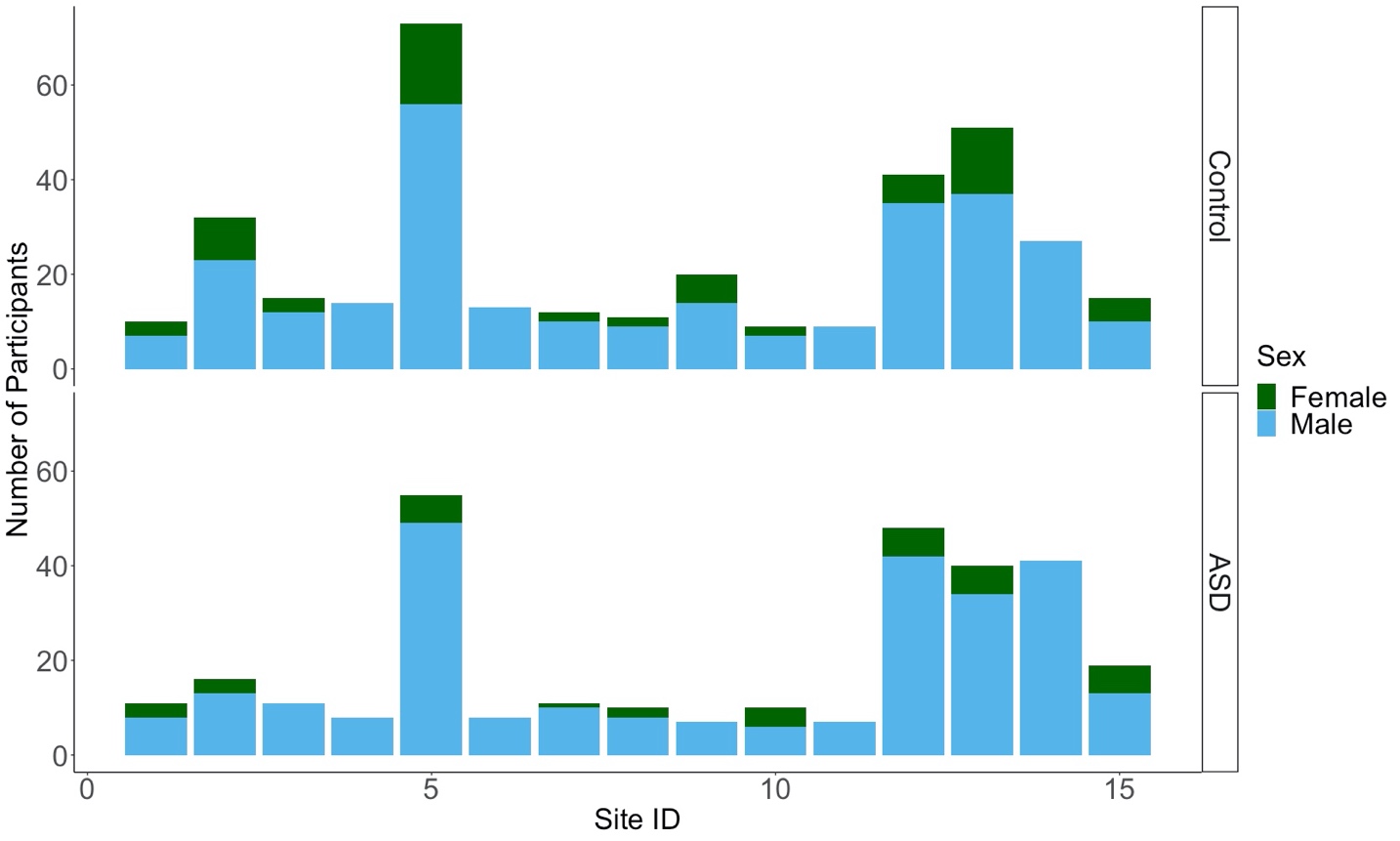


**Figures S12. Distribution of Participants by Scanner Site, Sex, and Group (Autism Spectrum Disorder (ASD) & Controls)**. Circles represent the brain volume of each participant. (1 = California Institute of Technology; 2 = Kenny Krieger Institute; 3 = University of Leuven Sample; 4= Ludwig Maximilians University Munich; 5 = NYU Langone Medical Center; 6 = Oregon Health and Science University; 7= Olin, Institute of Living at Hartford Hospital; 8= University of Pittsburgh School of Medicine; 9 = San Diego State University; l0 = Stanford University; 11 = Trinity Center for Health Sciences; 12 = University of California Los Angeles; 13 = University of Michigan Sample; 14 = University of Utah School of Medicine; 15 = Yale Child Study Center).


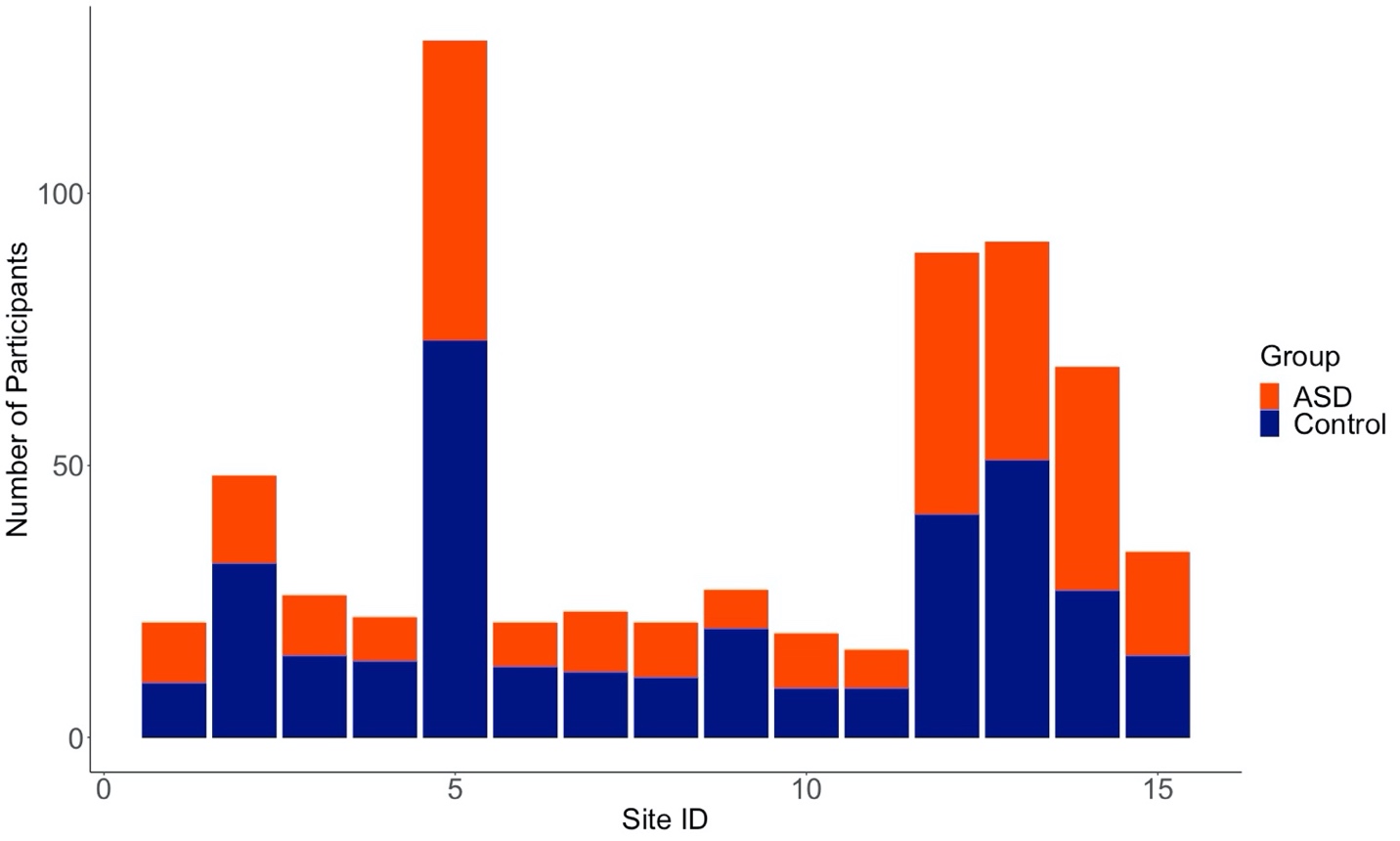


**Figures S13. Distribution of Participants by Scanner Site and Group (Autism Spectrum Disorder (ASD) & Controls)**. Circles represent the brain volume of each participant. (1 = California Institute of Technology; 2 = Kenny Krieger Institute; 3 = University of Leuven Sample; 4= Ludwig Maximilians University Munich; 5 = NYU Langone Medical Center; 6 = Oregon Health and Science University; 7= Olin, Institute of Living at Hartford Hospital; 8= University of Pittsburgh School of Medicine; 9 = San Diego State University; l0 = Stanford University; 11 = Trinity Center for Health Sciences; 12 = University of California Los Angeles; 13 = University of Michigan Sample; 14 = University of Utah School of Medicine; 15 = Yale Child Study Center).


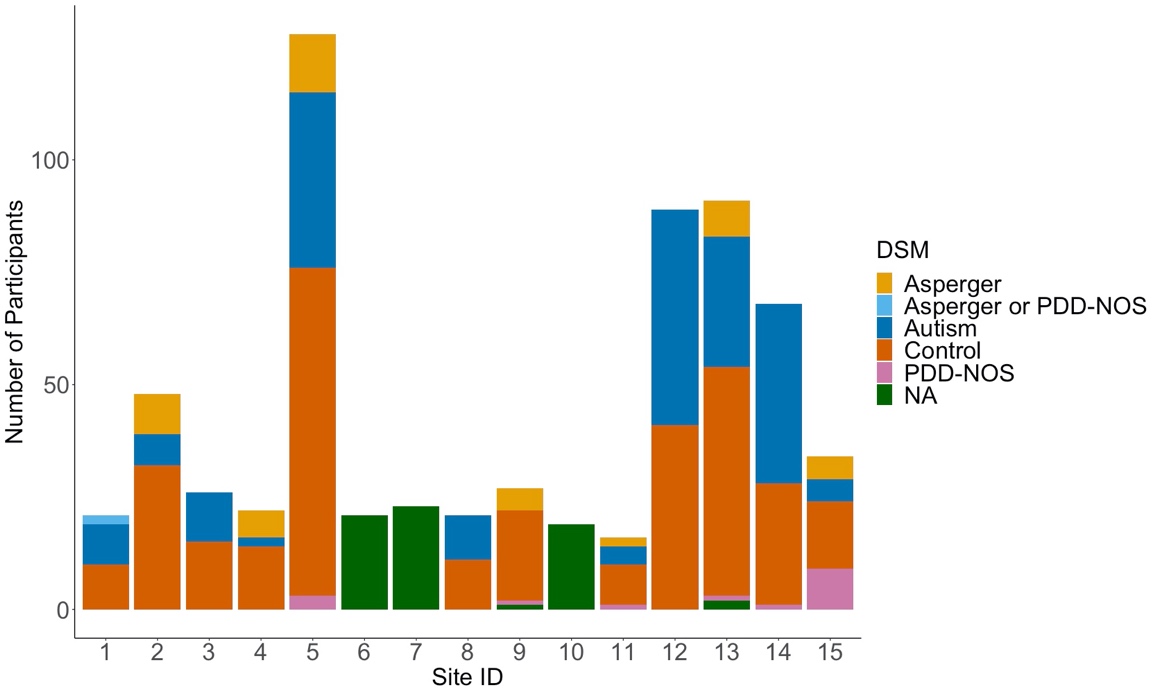


**Figures S13. Distribution of Participants by Scanner Site and DSM-IV-TR diagnostic when available. DSM-IV-TR:** fourth and text revised edition of the Diagnostic and Statistical Manual of Mental Disorder. ASD: Autism Spectrum Disorder. PDD-NOS: Pervasive Developmental Disorder-Not Otherwise Specified. NA: DSM-IV-TR not available. Circles represent the brain volume of each participant. (1 = California Institute of Technology; 2 = Kenny Krieger Institute; 3 = University of Leuven Sample; 4= Ludwig Maximilians University Munich; 5 = NYU Langone Medical Center; 6 = Oregon Health and Science University; 7= Olin, Institute of Living at Hartford Hospital; 8= University of Pittsburgh School of Medicine; 9 = San Diego State University; l0 = Stanford University; 11 = Trinity Center for Health Sciences; 12 = University of California Los Angeles; 13 = University of Michigan Sample; 14 = University of Utah School of Medicine; 15 = Yale Child Study Center).

1. **Power Analyses**

***Multiple Group Confirmatory Factor Analysis (MGCFA).***

Researchers suggest that the selection of a minimum sample size in factor analysis that provides a factor solution in agreement with the population structure from which the sample was taken depends on the number of observed variables (e.g. 22 regional volumes), factors (e.g. here, Total Brain Volume, TBV), number of variables per factor (e.g. here, 22 regional volumes), and the size of the communalities (i.e. the extent to which an observed variable correlates with the factor, which is small from 0.2 to 0.4, wide from 0.2 to 0.8, high from 0.6 to 0.8; Gaskin & Happell, 2014; Mundfrom, Shaw, & Ke, 2005).

To assess the size of the communalities, the R^2^ obtained from the lavInspect function (Rosseel, 2012) was inspected in each group and sample. While some regional volumes with very low communalities (<0.02) significantly loaded on the latent construct, the left accumbens and right amygdala did not load significantly for the ASD group in the sample of boys aged 6 to under 12 years old (R^2^ = 0.228, p = 0.185, R^2^ = 0.436, p = 0.76) and of boys with an FSIQ < median (R^2^ = 0.100, p = 0.164; R^2^ = 0.195, p = 0.055). Although the insignificance of these slopes (or loadings) suggests these regional volumes do not explain a proportion of variance in TBV in ASD individuals, these variables were maintained in the MGCFA models across samples since the right amygdala and left accumbens significantly contributed to explaining variance in TBV in controls. Considering the presence of few low communalities and that most communalities were wide but averaged around 0.4, we identified the level of communality as low to be conservative, requiring more participants. Thus, based on the simulation tables provided by Mundform and colleagues (2005), the MGCFA samples included in the current study were sufficiently large to obtain a factor solution in agreement with the population structure from which the sample was taken, as a minimum of 50 participants per group was required.

Yet, Mundform and colleagues’ (2005) criteria do not provide information on the power of our MGCFAs to detect group differences in allometric scaling (i.e. slopes or loadings) and volumes adjusted for allometric scaling (i.e. intercepts). To our knowledge, there are currently no packages developed to conduct power analyses to either detect the effect size of the group difference in slopes or intercepts with a predetermined sample or identify the number of participants required to observe a predetermined effect size of slope or intercept group differences, while including correlated residuals.

***Linear Mixed Effects Models (LMEMs)***.

Power analyses for LMEMs were conducted with the simr package (Green & MacLeod, 2016). Confidence intervals were set to 95% and power estimations were based on a 1000 simulations. Power analyses were only run on significant group main effects and interactions.

1. **MGCFA & LMEMs Assumptions**

***Univariate & Multivariate Normality.*** Univariate Normality was established by visually inspecting histograms of continuous variables (regional volumes, TBV, age, and FSIQ). Age and FSIQ were skewed and normal after scaling based on visual inspection of histograms. Raw volume values were normally distributed and became skewed after log10 transformation.

Univariate outliers were responsible for volumetric data non-normality based on visual comparison of brain volume histograms with and without outliers. Univariate outliers were identified separately in each group as the raw volume values that lied 1.5 times outside the interquartile range obtained from boxplots with ggplot2 (Wickham, 2016). Multivariate normality was examined in the LMEMs using Cook’s D (distance) with multivariate outliers defined as individuals with a Cook’s D 5 times greater than the mean. To correct for non-normality in the LMEMs, LMEMs were run once with outliers and once without univariate and multivariate outliers.

To correct for non-normality in MGCFAs, we ran the analyses with the MLR (Maximum Likelihood Robust) estimator provided by the lavaan package (Rosseel, 2012), which provides maximum likelihood parameters estimates with standardized errors and a chi-square test statistic that is robust to non-normality by implementing a mean adjustment.

***Multicollinearity***. Multicollinearity was examined with a Pearson’s Bivariate Correlation among all independent variables in the entire sample and in each group (Figures *S14-16*). Collinearity between observed variables is expected in the MGCFA since they are to load on the same factor. In LMEMs, multicollinearity between continuous variables was investigated with Pearson’s Bivariate Correlation and group differences in correlations investigated with the Fisher r to z transformation (Table *S5*). Although TBV and FSIQ were significantly correlated in the entire sample ( r = 0.17, p = 1.14 x 10^-5^) due to its significant correlation in the control but not the Autism Spectrum Disorder (ASD) group, FSIQ and TBV were maintained together in the LMEMs since correlation was weak. Although TBV and age (linear and quadratic) were significantly correlated in the entire sample (r = 0.18, p = 4.42 x 10^-6^ and r = 0.16, p = 1.65 x 10^-5^ respectively) due to its significant correlation in the ASD but not the control group, age and TBV were maintained together in the LMEMs since correlation was weak. The same reasoning was applied for the correlation between age (linear and quadratic) and FSIQ (r = 0.10, p = 0.009 and r = 0.12, p = 0.002, respectively).

**Heteroscedasticity.** Based on visual inspection of the residual plots of our models, residuals similarly deviated from the predicted values of the LMEMs models. Although some data points appeared as outliers, homoscedasticity was generally met across LMEM models. These outliers disappeared in the LMEMs conducted without multivariate and univariate outliers.


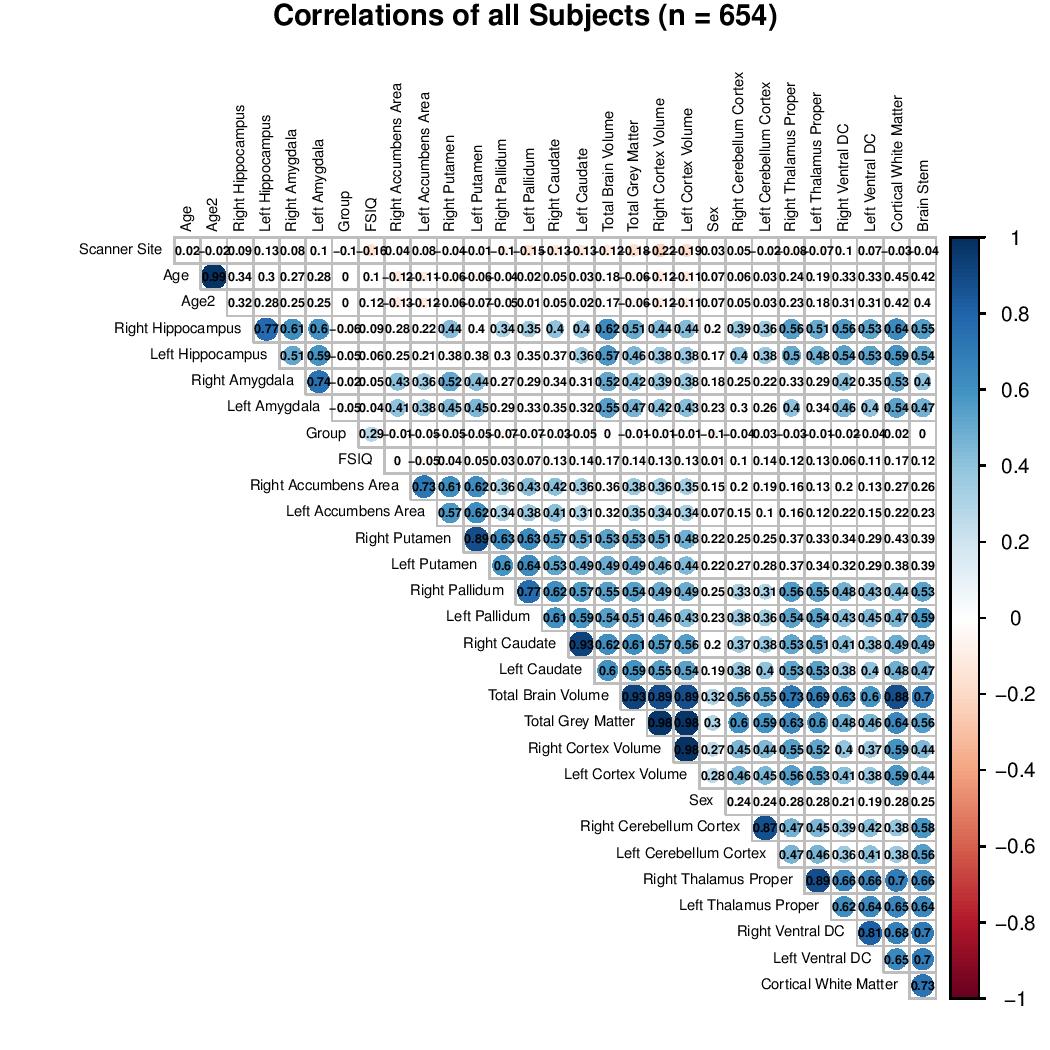


**Figure S14.** Correlation Matrix of all Cerebral Volumes, Age at Scan, Age2 (Quadratic Age), Sex, Diagnostic Group, and Full Scale Intelligence Quotient in the Entire Sample. (Correlations coefficients are in black. Significant correlations (p< 0.05) have bluer hues, indicating positive correlations, or reddish hues, indicating negative correlations. Group: Autism Spectrum Disorder or Control. DC: Diencephalon. FSIQ: Full Scale Intelligence Quotient.)

**
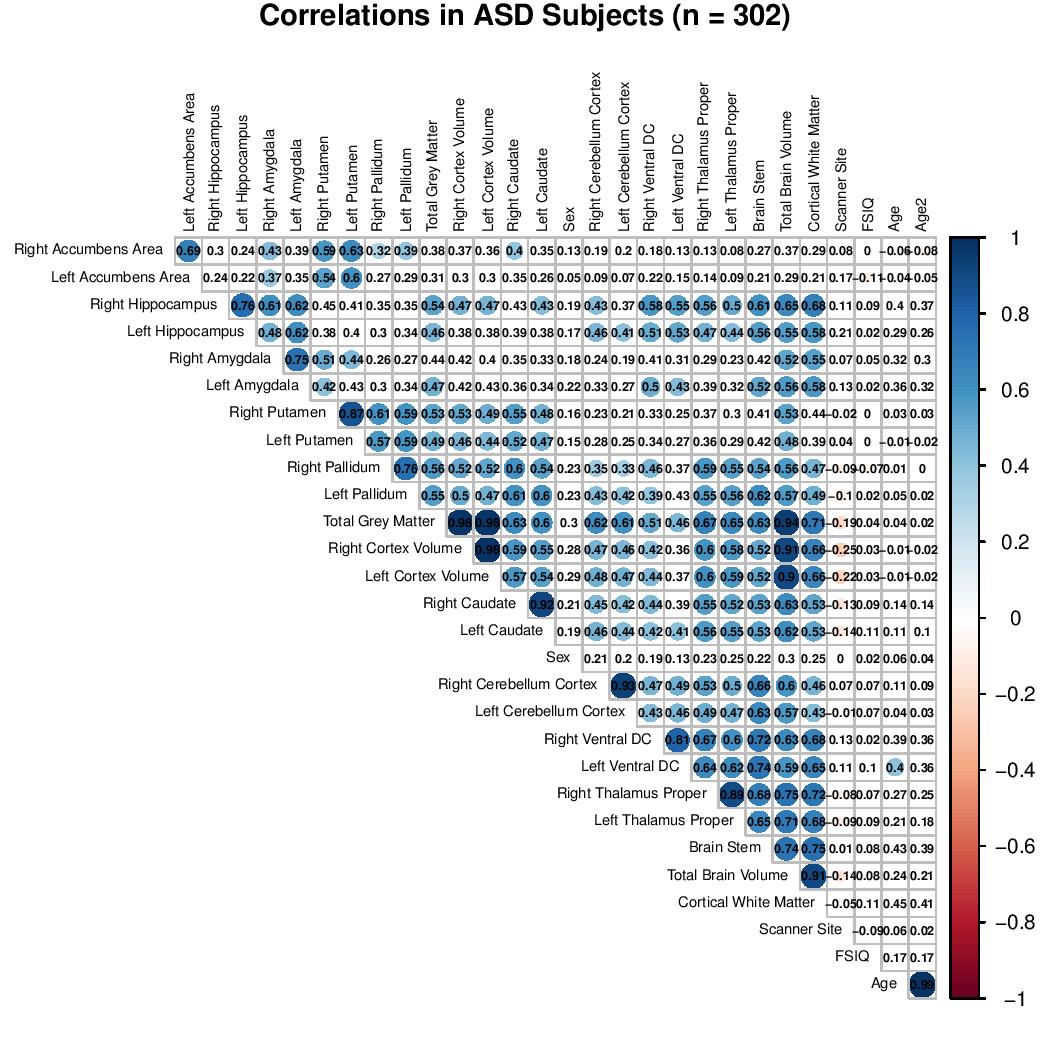
**

**Figure S15.** Correlation Matrix of all Cerebral Volumes, Age at Scan, Age2 (Quadratic Age), Sex, Diagnostic Group, and Full Scale Intelligence Quotient in Autism Spectrum Disorder (ASD) Participants. (Correlations coefficients are in black. Significant correlations (p< 0.05) have bluer hues, indicating positive correlations, or reddish hues, indicating negative correlations. DC: Diencephalon. FSIQ: Full Scale Intelligence Quotient.)

**
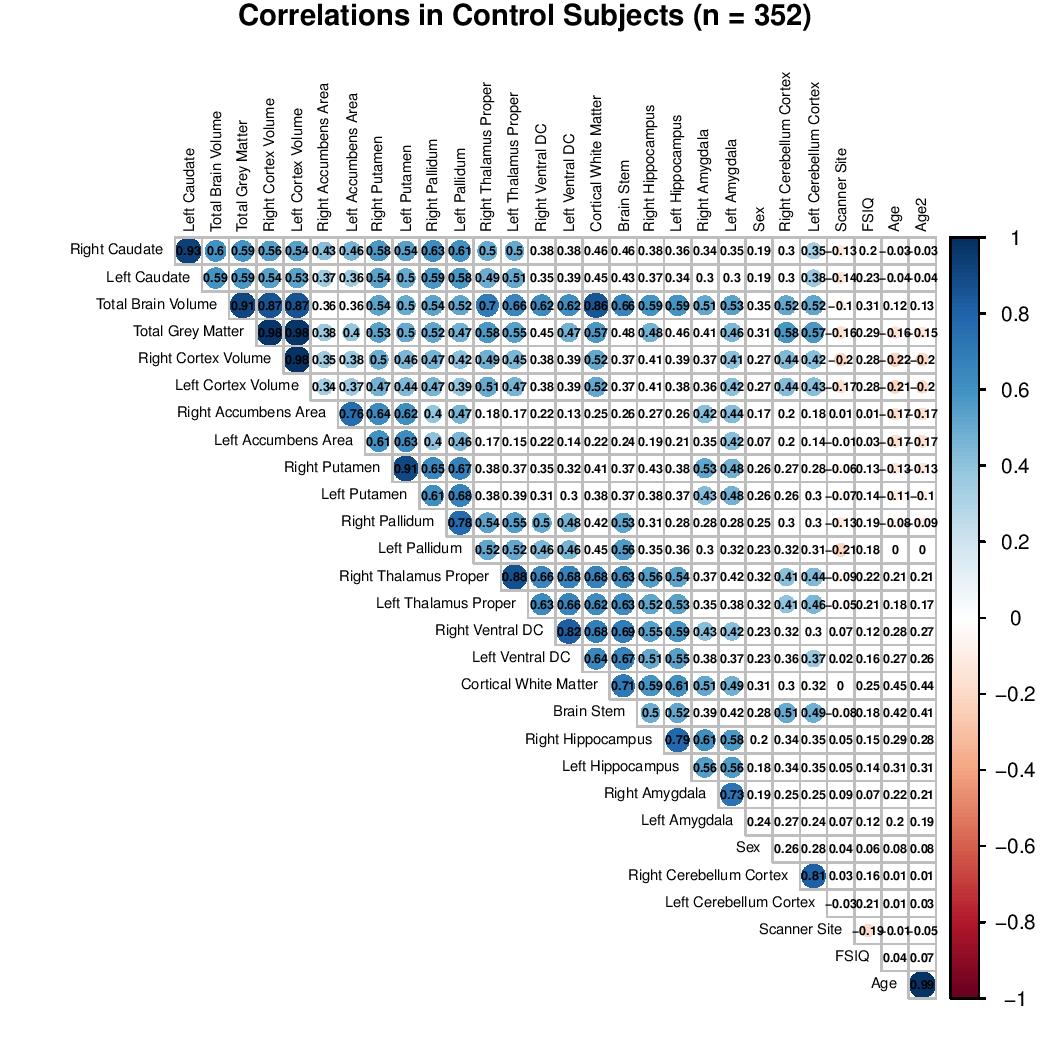
**

**Figure S16.** Correlation Matrix of all Cerebral Volumes, Age at Scan, Age2 (Quadratic Age), Sex, and Full Scale Intelligence Quotient in Control Participants.

(Correlations coefficients are in black. Significant correlations (p< 0.05) have bluer hues, indicating positive correlations, or reddish hues, indicating negative correlations. DC: Diencephalon. FSIQ: Full Scale Intelligence Quotient.)

1. **Replication of Zhang and Colleagues (2018): Methodology**

Since the latest study examining the age and sex effects on the subcortical correlates of autism with the ABIDE I previously conducted LMEMs (1) without TBV Adjustment and (2) with linear TBV adjustment, we replicated Zhang and colleagues’ (2018) statistical analyses as well as conducted additional LMEMs (3) with allometric scaling as the TBV adjustment to validate their findings and identify if reported volumetric group differences depend on the presence and the type of adjustment for individual differences in TBV.

As in Zhang and colleagues (2018) analyses in SPSS, the dependent variables in the LMEMs were the Cortical WM Volume, Total GM Volume, the Caudate Nucleus, the Amygdala, the Hippocampus, the Thalamus, the Pallidum, the Putamen, and the Accumbens. Fixed effects differed based on the type of adjustment technique, as described below. Scanner site was always included as a random intercept and subject as a random intercept when hemisphere was included in the LMEMs model. Dependent and independent variables were entered in the models as raw values except for age (linear and quadratic), which was centered (i.e. demeaned). Significant group main effects and interactions were reported and compared across LMEMs with varying adjustment techniques. As in Zhang and colleagues (2018) p values were not adjusted for multiple comparison.

**LMEMs without TBV Adjustment.** Fixed effects were sex, age (quadratic or linear), hemisphere (except for Cortical WM and Total GM volumes), and group (ASD and Controls). Two replication strategies were put into place: a “result replication” and a “methodological replication”. In the “result replication”, models were identified based on the significant interactions reported by Zhang and colleagues (2018) to compare effect sizes even if group interactions and main effects were not significant in our sample. In the “methodological replication”, final LMEMs were identified using Zhang and colleagues’ (2018) model simplification technique, which corresponded to maintaining main effects in the model and sequentially removing all non-significant interactions (p > 0.05) from the model.

**LMEMs with linear TBV Adjustment.** As in Zhang and colleagues (2018) analyses, TBV was added as a covariate to the LMEMs identified with the “result replication” and “methodological replication” techniques. Although Zhang and colleagues (2018) ran models with and without TBV as a covariate, they commented on whether results were similar after covarying for TBV without providing any statistics (i.e. effect sizes and p values).

**Comparing LMEMs with the lack of and differing TBV Adjustment Techniques.** All brain volumes were log 10 transformed prior to scaling. LMEMs identified with the “result replication” and “methodological replication” techniques were run with the interaction of group by log10 (TBV).

1. **MGCFA Results**

**Correlated Residuals.**

Correlated residuals represent correlations between observed variables not explained by the latent construct and improve model fit by allowing observe variable error terms to covary when they are part of the same factor. The correlated residuals included in the entire sample and each subsample slightly differed, since the same correlated residuals across samples did not enable acceptable model fit (Table *S14*). For instance, 76% of the correlated residuals in the boys from 6 to under 12 years old sample were included in the Entire Sample and 74% of the correlated residuals in the Entire Sample were included in the sample of boys from 6 to under 12 years old.

**Model Fit Indices.**

Fit indices overall indicated good fit across samples. However, the robust TLI was slightly under < 0.95 for control boys with an FSIQ > 107.8 and the robust RMSEA was slightly over < 0.06 for ASD boys aged 6 to under 12 years old and ASD and control boys with an FSIQ > 107.8. Yet, considering that the majority of model fit indices indicated good fit, that previous studies accept fit indices values close to the model fit cutoff values (Xu & Tracey, 2017), and that adding more correlated residuals to improve fit resulted in a non-positive definite residual covariance matrix (making results not interpretable), these model fit indices were judged as acceptable (Table *S15*).

***TBV Group Differences.*** In the MGCFA, TBV did not differ between individuals with and without ASD in the entire sample (ß = 0.03, SE = 0.06, p = 0.437), in boys from 6 to under 12 years old (ß = -0.08, SE = 0.10, p = 0.260), in boys from 12 to under 20 years old (ß = 0.03, SE = 0.09, p = 0.441), and in boys with an FSIQ > 107.8 (ß = -0.04, SE = 0.11, p = 0.451).

***Regional Allometric Scaling Group Differences.*** Considering the exploratory result that allometric scaling differed between groups in boys from 12 to under 20 years old and in boys with an FSIQ under the median, we expected an interaction of TBV by group by sex by age and of TBV by group by sex by FSIQ in the model with TBV by group by age by sex by FSIQ as fixed effects and scanner site as random intercept in the entire sample. However, neither interaction was significant (ß = -0.03, SE = 0.20, p = 0.982, and ß = -0.07, SE = 0.25, p = 0.931, respectively).

*Boys from 12 to under 20 years old.* Table *S16.A.* shows that the robust CFI and robust RMSEA fit indices were invariant across models according to Chen’s metric invariance cutoffs (|ΔCFI| >.005 & |ΔRMSEA| ≥.010. Although the χ2 metric invariance test implied that groups differ in terms of allometry in the left amygdala and brain stem, the corresponding LMEMs were consistent with our interpretation of the MGCFA results suggesting the absence of an allometric scaling group difference in these volumes (respectively, ß = -0.04, SE = 0.09, p = 0.693; ß = 0.09, SE = 0.09, p = 0.322). Figure *S17* shows the allometric scaling group difference in the right hippocampus without outlier and comorbidity removal.

*Boys with an FSIQ > median (107.8).* Table *S16.B.* shows that the robust CFI and robust RMSEA fit indices were invariant across models according to Chen’s metric invariance cutoffs (|ΔCFI| >.005 & |ΔRMSEA| ≥.010. The χ2 metric invariance test implied that groups also differ in terms of allometry in the left hippocampus, caudate, pallidum and ventral diencephalon. These group difference sin allometric scaling were additionally reported in the corresponding LMEMs: left hippocampus (ß = -0.29, SE = 0.10, p = 0.009), caudate (ß = -0.36, SE = 0.12, p = 0.006), pallidum (ß = -0.26, SE = 0.10, p = 0.023), and the right ventral diencephalon (ß = -0.28, SE = 0.10, p = 0.017). However, they were no longer significant after including medication as a covariate and removing outliers and comorbidities (hippocampus, ß = -0.09, SE = 0.10, p = 0.765; caudate, ß = -0.01, SE = 0.11, p = 0.429; pallidum, ß = 0.08, SE = 0.11, p = 0.607; and ventral diencephalon, ß = - 0.23, SE = 0.12, p = 0.143). Figure S18 shows the allometric scaling group difference in the left accumbens without outlier and comorbidities and Table *S18* the linear Mixed Effects Model with Quadratic Age.


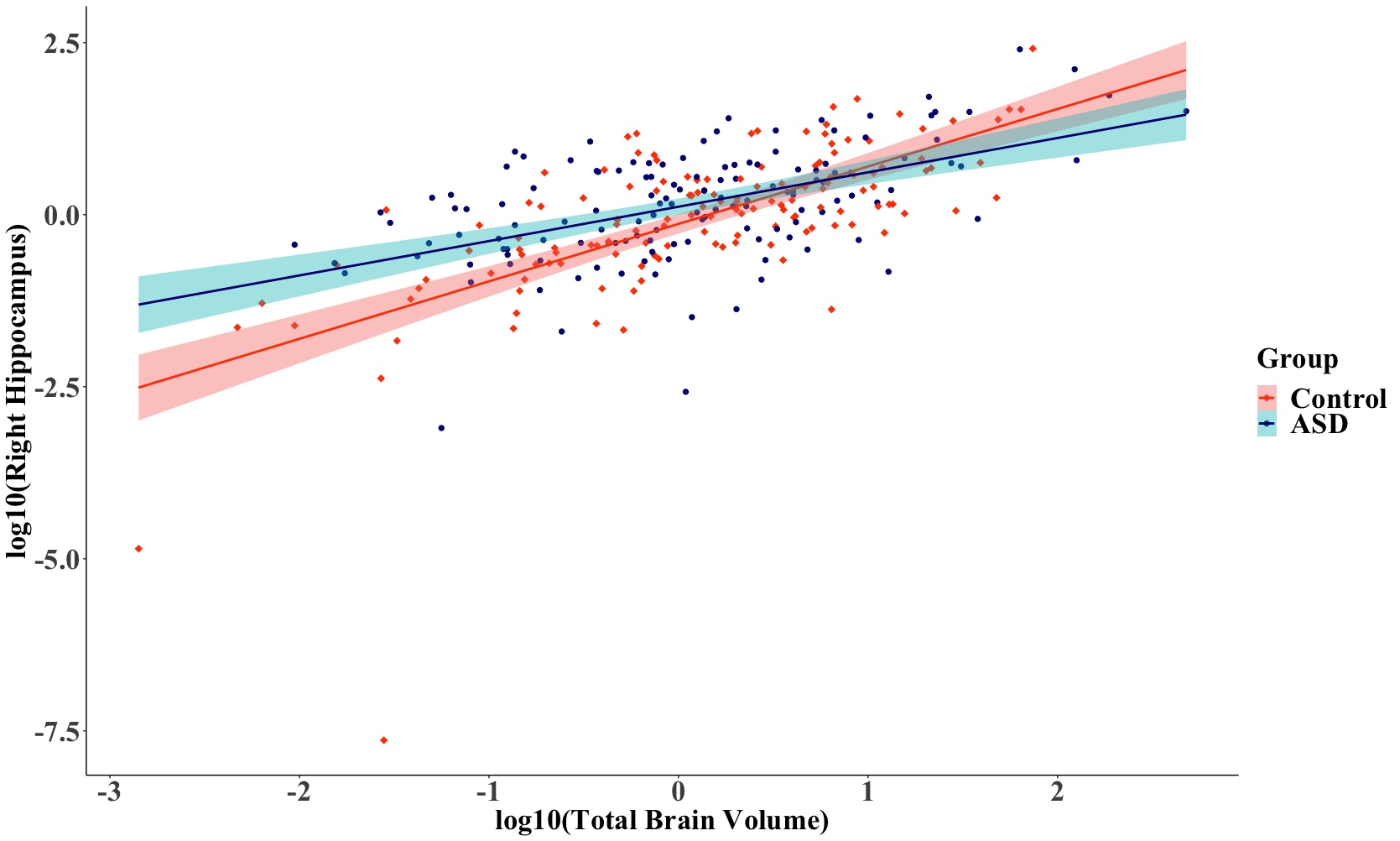


**Figure S17.** Relationship between the right hippocampus and total brain volume across groups before (N_Control_ = 141, N_ASD_= 138) outlier and comorbidity removal in boys aged 12 to under 20. ASD, Autism Spectrum Disorder. 95% confidence region are given by group. Volumes are log transformed and scaled.


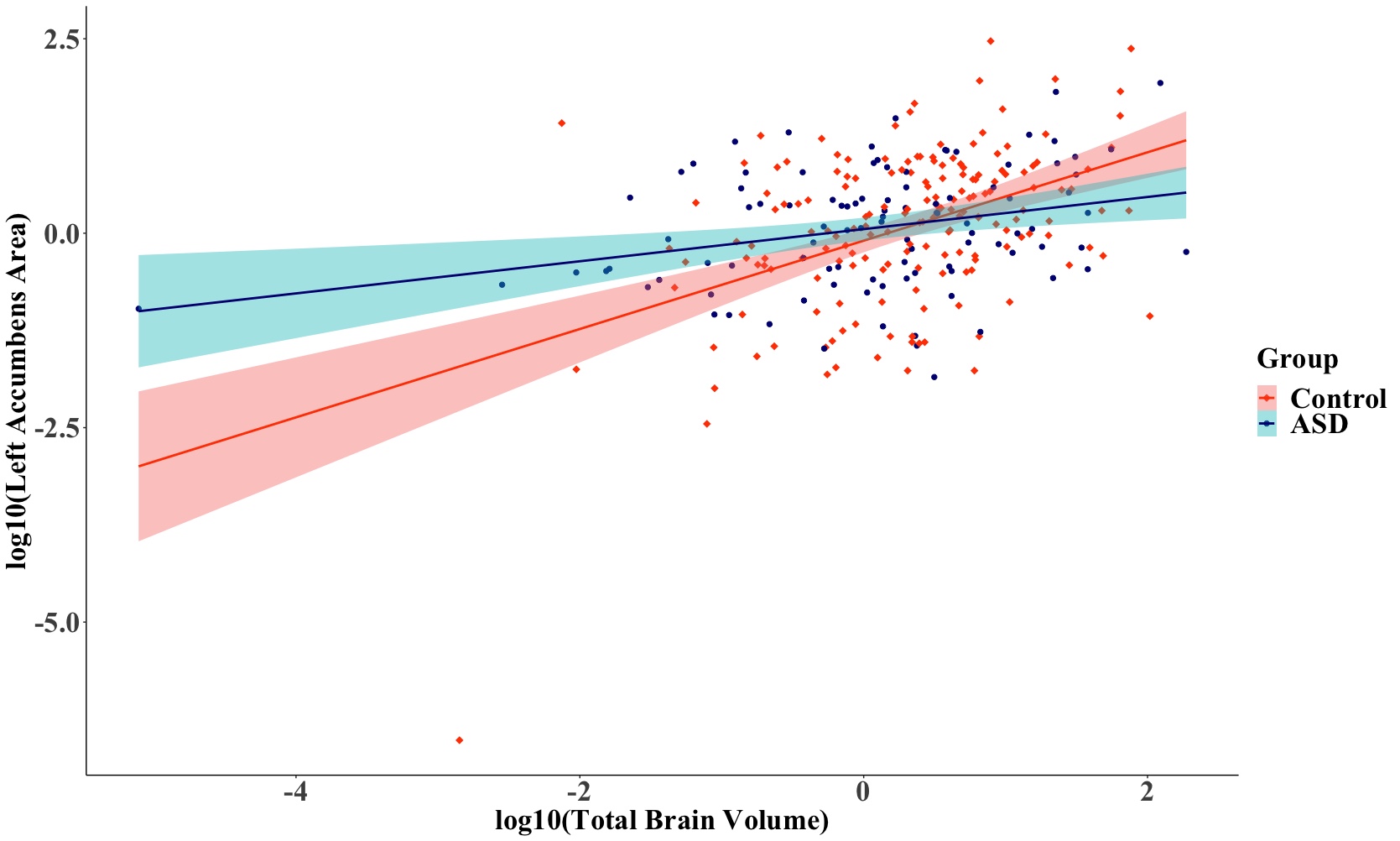


**Figure S18.** Relationship between the left accumbens and total brain volume across groups before (N_Control_ = 174, N_ASD_= 100) outlier and comorbidity removal in boys with a Full Scale Intelligence Quotient < median (107.8). ASD, Autism Spectrum Disorder. 95% confidence region are given by group. Volumes are log transformed and scaled.

1. **Replication Results**

The tables S19 to S27 compare the present study’s replication of Zhang and colleagues’ (2018) Linear Mixed Effects Models (LMEMs) without adjusting for TBV differences to Zhang and colleagues (2018) findings and to LMEMs with Linear TBV Adjustment and with Allometric Scaling TBV Adjustment. The “result replication” models were identified based on the significant interactions reported by Zhang and colleagues (2018) to compare effect sizes even if group interactions and main effects were not significant in our sample. The “methodological replication” models were identified using Zhang and colleagues’ (2018) model simplification technique, which corresponded to maintaining main effects in the model and sequentially removing all non-significant interactions (p > 0.05) from the model. Group levels were 1: Controls and 2: ASD and sex levels were 1: Female and 2: Male. Raw values were included in the models, age was centered, and scanner site and hemisphere (except for total grey volume and cortical white matter volume) were added as random intercepts.

**Supplemental Information Bibliography**

Gaskin, C. J., & Happell, B. (2014). On exploratory factor analysis: A review of recent evidence, an assessment of current practice, and recommendations for future use. *International Journal of Nursing Studies*, *51*(3), 511–521. https://doi.org/10.1016/j.ijnurstu.2013.10.005

Green, P., & MacLeod, C. J. (2016). simr: An R package for power analysis of generalised linear mixed models by simulation. *Methods in Ecology and Evolution*, *7*(4), 493–498. https://doi.org/10.1111/2041-210X.12504

Mundfrom, D. J., Shaw, D. G., & Ke, T. L. (2005). Minimum Sample Size Recommendations for Conducting Factor Analyses. *International Journal of Testing*, *5*(2), 159–168. https://doi.org/10.1207/s15327574ijt0502_4

Rosseel, Y. (2012). lavaan: An R Package for Structural Equation Modeling. *Journal of Statistical Software*, *48*(2), 1–36.

Wickham, H. (2016). *ggplot2: Elegant Graphics for Data Analysis*. Retrieved from http://ggplot2.org

Xu, H., & Tracey, T. J. G. (2017). Use of Multi-Group Confirmatory Factor Analysis in Examining Measurement Invariance in Counseling Psychology Research. *The European Journal of Counselling Psychology*, *6*, 75–82. https://doi.org/10.5964/ejcop.v5i2.120

Zhang, W., Groen, W., Mennes, M., Greven, C., Buitelaar, J., & Rommelse, N. (2018). Revisiting subcortical brain volume correlates of autism in the ABIDE dataset: Effects of age and sex. *Psychological Medicine*, *48*(4), 654–668. https://doi.org/10.1017/S003329171700201X
